# Exploring Support Preferences for Young Women Who Self-Harm: A Qualitative Study

**DOI:** 10.3390/ijerph22040587

**Published:** 2025-04-09

**Authors:** Demee Rheinberger, Smrithi Ravindra, Aimy Slade, Alison L. Calear, Amy Wang, Brittany Bunyan, Helen Christensen, Isabel Mahony, Isabella Gilbert, Katherine Boydell, Lorna Hankin, Samantha Tang

**Affiliations:** 1 Black Dog Institute, UNSW Sydney, Sydney, NSW 2033, Australia; smrithi.ravindra@blackdog.org.au (S.R.); aimy.slade@blackdog.org.au (A.S.); h.christensen@blackdog.org.au (H.C.); k.boydell@blackdog.org.au (K.B.); samantha.tang@blackdog.org.au (S.T.); 2 Centre for Mental Health Research, Australian National University, Canberra, ACT 2601, Australia; alison.calear@anu.edu.au; 3 Discipline of Clinical Psychology, Graduate School of Health, University of Technology Sydney, Ultimo, NSW 2007, Australia; 4 Faculty of Medicine and Health, UNSW Sydney, Sydney, NSW 2033, Australia

**Keywords:** self-harm, young women, support needs, qualitative research

## Abstract

Rates of self-harm among young women have been increasing in recent years across multiple high-income nations. Given the negative outcomes associated with self-harm, it is essential that young women who engage in self-harm receive the support that best addresses their specific needs. The aim of the current study is to explore support preferences among Australian young women with a history of self-harm. Semi-structured interviews were conducted with 27 young women (*M* = 20.9, *SD* = 2.1) with a history of self-harm, recruited through social media posts. Interviews were audio-recorded and transcribed verbatim. Data were analysed using a Reflexive Thematic Analysis approach. Thematic analysis of the data identified themes pertaining to the following: (1) the types of support young women want around self-harm, and (2) how young women would like to receive support for self-harm. Regarding the former, participants expressed wanting information about self-harm and self-harm interventions, education about self-harm for those around them, age-specific mental health education, and alternative coping strategies. Some participants expressed not wanting any support. Regarding the latter, participants wanted self-harm information to be provided by health professionals and in school settings, and preferred for information to be provided in written or digital forms. The findings highlight the need to improve access to formal mental health support among young women, the importance of young people being able to access support confidentially, and the need for young peoples’ caregivers and health professionals to be educated about self-harm. Additionally, the findings support a role for schools in providing information about mental health and available support.

## 1. Introduction

In Australia, self-harm hospitalisation rates of young women have increased steadily from 2008–2009 to 2022–2023 [1], but rates of self-harm not resulting in hospitalisation are estimated to be much higher, with the Australian Bureau of Statistics suggesting that 25% of all Australian young women between 16 and 24 years of age have engaged in self-harm in their lifetime [2]. Defined as deliberately injuring or poisoning oneself regardless of suicidal intent [3], self-harm impacts upon a range of adverse outcomes relating to mental health disorders, psychological distress, substance use, employment, education attainment, and broader quality of life [4,5,6]. The rise in self-harm rates among young people is also particularly concerning given its association with a higher risk of suicide [7,8].

In conjunction with pharmacological treatments, clinical guidelines recommend clinic-based psychosocial interventions such as dialectical behavioural therapy, cognitive behavioural therapy, and family intervention for the prevention or reduction of self-harming behaviours in young people [9,10,11,12]. These interventions address the well-established links between self-harm and mental health difficulties, adverse childhood experiences, emotional regulation, family conflict, and poor social support [13,14]. They are generally delivered by mental health professionals (including psychiatrists, psychologists, counsellors, or other clinicians) and assist young people in improving their coping skills, social connectedness, mood tolerance, and self-esteem, in addition to managing any co-existing mental health disorders [9].

However, despite the effectiveness of such interventions in reducing self-harming behaviour [12], young people rarely seek help for their self-harming behaviour from mental health professionals [15]. Although young Australians who engage in self-harm report having a higher perceived need for help-seeking, this does not necessarily translate into higher service utilisation [16]. This is consistent with findings from a retrospective cohort study conducted by Wong et al. [17], which reported a recent decrease in hospital admissions for self-harm among young people across 10 countries worldwide, despite an increase in the proportion of children and adolescents presenting with self-harm.

Moreover, an Australian community-based survey of young adults noted that 40% of those who were engaged in therapy and experienced suicidal ideation did not disclose the suicidal ideation to their clinician [18]. Stigma has been identified as an underlying factor when young people make help-seeking decisions [19], with higher levels of stigma reducing help-seeking intentions [20]. Given the pervasive stigma surrounding self-harm, it is also possible that young people do not discuss their self-harming behaviours with their health professionals. Instead, young people describe a preference to seek help for self-harm from informal support networks, namely caregivers, teachers and peers [21,22], and other sources of information such as the internet, helplines, pamphlets, and websites [23]. It is unlikely that parents and peers have the appropriate knowledge and skills to support young people, including how to discuss self-harm safely and how to connect young people to relevant professional support and/or resources [24,25]. While extant research [26,27,28] highlights the potential for digital technologies such as online or mobile-based interventions in delivering the necessary mental healthcare to aid young people who self-harm [26,27,28], little is known about what information young people would like to receive from such resources.

There is limited evidence regarding the support preferences of young women who engage in self-harm, and further exploration is warranted. To this end, it is necessary to understand the type of information young people would find helpful in relation to their self-harm behaviour. To our knowledge, only one study to date [29] has investigated this question, with the authors finding that young people appreciate having a ‘toolbox of resources’, or a diverse set of strategies and information to draw upon for their understanding of self-harm and for momentary distress management. There is still uncertainty, however, regarding the specific support information that young people want in these toolboxes and their preferred delivery format. Co-designing these resources with young people is key to addressing these gaps and ensuring that the support available is appropriate for the target population [30,31]. However, few studies have utilised this important lived experience perspective, and there are, to the author’s knowledge, no studies that explore the perspective of young women specifically, despite the higher rates of self-harm in this population. Thus, using qualitative methodologies, the current study aimed to explore the types of support young women with a history of self-harm want and how they would like this support to be delivered.

## 2. Materials and Methods

### 2.1. Study Setting

This qualitative study is part of a larger study exploring the experience of self-harm in young Australian women, the findings from which will be reported across numerous papers. Study approval was provided by the UNSW Human Research Ethics Committee (HC230437).

### 2.2. Recruitment

Participants were recruited between October 2023 and November 2023 via purposive sampling, utilising advertisements on a well-known mental health agency’s (the Black Dog Institute) social media platforms (Facebook and Instagram). Individuals were directed to the study website with information about the study and a link to the participant information sheet, consent form, and an eligibility screener.

Individuals were eligible to participate if they identified as female, were aged between 16 and 24 years (inclusive), had a history of or experienced current self-harm (defined as intentionally causing pain or damage to their own body, either with or without suicidal intent), spoke English, and lived in Australia. A Gillick competency assessment [32] was used to assess whether minors (i.e., participants aged 16–17 years) adequately understood the study requirements and were therefore competent to participate without the consent of a parent or guardian. The Gillick competency assessment was administered during the screening process and involved answering five questions about the study. Participants who failed the Gillick competency assessment were deemed ineligible to participate. There were no other exclusion criteria. Ineligible participants were directed to a page containing support helplines. Eligible participants were asked to provide contact and demographic information (such as employment status, sex at birth, ethnicity, history of a mental health diagnosis) and were contacted by a member of the research team to schedule the interview.

### 2.3. Data Collection

Twenty-seven one-on-one interviews were conducted by five Clinical Psychology Masters students via an online, secure video conferencing software during November and December 2023. The interviewers were trained to conduct interviews by the lead author, who has extensive experience conducting interviews with individuals who have engaged in self-harm or experienced suicide crisis, to ensure that the interviewers understood how to conduct the interviews and to facilitate consistency across all interviews. The interviewers were experienced in engaging with individuals who have self-harmed in a clinical context and had received appropriate clinical training to assess for emotional distress and risk of harm prior to study involvement. Additionally, all the interviewers were provided clinical supervision by a registered clinical psychologist.

A safety protocol was in place to respond in case of a participant’s disclosure of imminent risk of suicide. This involved immediate intervention by a supervising clinical psychologist to ensure participant safety. The interviewers utilised their clinical judgement in cases of participant distress during the interview and would cease the interview and connect the participant through to appropriate clinical support if necessary. Alternatively, interviewers would offer a break, or to reschedule the interview to a later date. No participants indicated imminent suicide risk or became so distressed that interviews were ceased.

Interviews ranged from 41 to 106 min in length (average: 70 min). The semi-structured interview guide included questions about self-harm history, stressors and motivations, help-seeking history, and service preferences (see Supplementary S1). The guide was developed collaboratively by researchers, clinical psychologists, postgraduate clinical psychology students, and individuals with lived experience of self-harm. Upon completion of the interview, participants were sent contact details for support helplines and were provided with an e-gift card for their participation. Interviews were audio recorded and sent to a secure third-party service for transcription. Interview recordings were reviewed by the lead author upon completion to assess for saturation of novel ideas [33]. The lead author kept a log of the novel ideas discussed during the interviews, and data collection ended once saturation was reached. No checking or validation from participants was conducted.

### 2.4. Analysis

This interpretive phenomenological study involved the analysis of interview data, guided by Reflexive Thematic Analysis (RTA) [34], using Nvivo software (version 12). Given the involvement of suicide/self-harm researchers and clinicians during data collection and analysis, RTA allowed for these perspectives to be integrated into the interpretation of the findings, creating more nuanced and complex insights. The analysis was led by the lead author (DR), a PhD candidate who has extensive experience conducting qualitative research investigating suicide and self-harm. The analysis was further supported by ST, a postdoctoral researcher and clinical psychologist with more than five years’ experience supporting individuals who engage in self-harm, as well as in conducting research into suicide and self-harm. After prolonged familiarisation with the data, which included listening to the audio recordings and repeated readings of transcripts, data relating specifically to the types of support participants would prefer for their self-harming behaviours was isolated from the interview data. Other data collected from the interviews will be reported elsewhere. Codes were then developed inductively through a process of line-by-line review of isolated dated from the interview transcripts and iteratively reviewed until a final set of codes was generated. These codes were then reviewed and grouped based on shared meaning, resulting in two themes which explored what support young people wanted to receive for their self-harm, and how they would like to receive it. All stages of the analysis involved regular discussions between DR and ST about the data and our interpretations. A detailed audit log and reflection journal was maintained to uphold rigour through the analysis process and to facilitate continued reflexivity.

## 3. Results

### 3.1. Participants

All 27 participants discussed the support they would like concerning self-harm. The average age of participants was 20.9 years (*SD* = 2.1, range = 18–24). All participants had engaged in self-harm on two or more occasions, and all had a mental health diagnosis, with depressive disorders (*n* = 21, 77.8%) and anxiety disorders (*n* = 17, 63.0%) being the most common. Twenty-one (77.8%) were employed part-time, and the remaining were unemployed (*n* = 6, 22.2%). No participants identified as Aboriginal or Torres Strait Islander. More detailed demographic information is presented in Table 1.

Participants began self-harming during adolescence (12–18 years) (*n* = 21, 77.8%) or childhood (6–11 years) (*n* = 5, 18.5%), and one participant (3.7%) started self-harming in young adulthood. Almost half of the participants (*n* = 13, 48.1%) said they were currently engaging in self-harm, eight (29.6%) had most recently self-harmed more than a year ago, and six (22.2%) had self-harmed within the last year.

### 3.2. Thematic Analysis

The thematic analysis resulted in two themes which spoke to young women’s support preferences in relation to self-harm: (1) what support they want to receive, and (2) how they would like to receive support (see Table 2).

#### 3.2.1. What Support Young Women Want Around Self-Harm

##### Information About Self-Harm

Participants wanted a plethora of information about self-harm. Young women spoke about previously receiving information about self-harm that was presented in a way that belittled or stigmatised self-harm. This was often inconsistent with their internal experience of significant emotional struggles. Participants also spoke about the focus on common ways of self-harming and feeling dismissed or ignored when their self-harm did not match with what is socially accepted. This stigma increased feelings of shame and made it difficult for some young women to reach out for help. Participants wanted information about the prevalence of self-harm and the different forms self-harm can take beyond more commonly recognised forms of self-harm (e.g., cutting) to help them feel less isolated.

“I just wish I knew that I didn’t have to be super, super sick or I don’t know, deeply depressed to deserve help. That was when I reached out, I was like, okay, now I can reach out. Now I’m actually struggling.”

Participant 23

“I think that a lot of what’s out there is that was really aimed at self-harm because of cutting or burning. But I think that self-harm is more broad than that, but that there’s not a lot of acknowledgement and often being dismissed by adults … they’re like, ‘oh, well she’s not cutting, so she can’t be that mentally unwell’.”

Participant 7

Young women also wanted more information about why people may start engaging in self-harm. The participants seemed overwhelmed by their experience and many struggled to understand why they had started to self-harm; they felt this information might help them to make sense of their own self-harm. Similarly young women also wanted information about how people feel prior to engaging in self-harm, so they could better recognise their own triggers and warning signs.

“I think it would be helpful if there was someone or something that helped me to understand more of why I was, I think at the start, the behaviour was quite impulsive, so I think it would’ve been helpful for me to understand why I was self-harming, and then support to I guess work through that.”

Participant 19

“Information that explained why I was so drawn to it, I didn’t know it. I didn’t really understand what I was feeling at the time. I had no insight. I just knew it made me feel better, but I didn’t know why. I think if resources were less stigmatizing and used less kind of floaty shades of grey language and were just outright, ‘Here’s why some people self-harm, here’s how you may feel after. Here’s why it’s not a healthy coping mechanism. Here are the long-term effects that are harmful’. If resources were just more upfront about it and spoke about it more openly, I think that would’ve really helped lessen the shame I felt and the pressure to hide it from people as well.”

Participant 20

Participants acknowledged that some information may encourage others to engage in self-harm behaviours, and were clear that they did not want information that would encourage others to start engaging in the behaviour. Many participants recognised that it was a tricky balance between helpful and risky information sharing.

“Don’t want to make it sound like it’s okay. I would hate for any young kids to fall into the groove that I’d fallen into. So practically speaking, how do you make it? ‘Please don’t do it. But also, if you do it, it’s okay’. I don’t know practically how to do that. I reckon it’s helpful for people to know where help is, but maybe [that] you don’t have to be unwell to get help.”

Participant 13

##### Information About Injury Management

Numerous young women also spoke about wanting information about how to care for themselves after a self-harm incident (e.g., wound management) to reduce the longer-term impact of their self-harming behaviour. Many reflected on the difficulty accessing this information and having to rely on others with a self-harm history to know how to care for their injuries.

“I think wound care is a big thing that is not addressed enough… I think also signs and symptoms of infections is probably another good one. I think even when to access help, what help you need for different levels of wounds. I feel like I know I’ve had friends call me being like; ‘what do I do when I need help? What do I need to do when this happens?’ So, things like that would be helpful.”

Participant 14

One participant spoke about receiving invalidating responses from hospital staff when she attended hospital for wound management. She felt that having the knowledge of how to care for the injuries would reduce young women’s exposure to stigmatising interactions.

“it’s important that you take care of your health, so you don’t have to go to the hospital and get that invalidation that you don’t want. So, I think particularly with methods like burning, I didn’t really know what I needed to do in order of safety. I knew that I should let them heal, but that’s also not what my routine was, was actually the opposite. So I just wish there was more non-judgmental access to information.”

Participant 17

##### Information for the People Around Them

Young women spoke of needing those around them (e.g., family members and friends) to be educated about self-harm, as these individuals often did not understand the nature of self-harm. This lack of understanding made it difficult for participants to receive support from others, or made them unwilling to seek support as they believed others would not understand their situation or needs. Young women spoke of loved ones using shame and misinformation to try to dissuade them from self-harm, which only reduced the likelihood that they would reach out for help again.

“So I think having more resources on being shaming your child or shaming your friend or whatever out of it isn’t actually helpful. I think a lot of people still use that angle of being like, oh, well, what if you get married one day and on your wedding day you’re going to have scars?”

Participant 3

Participants wanted those around them to understand why people engage in self-harm—that is, recognition that self-harm is often precipitated by negative experiences or emotional states. They also wanted others to understand the purpose and functions of self-harm, how to spot the signs of distress, how to have appropriate conversations about self-harm (e.g., non-shaming language), and to be more aware of the seriousness of self-harm. Many felt this would reduce the frequency of invalidating and stigmatising responses from others and may improve their likelihood of reaching out for help with self-harm and their underlying mental health concerns.

“I think just a more understanding of that from my personal support system would’ve probably, it would’ve helped a bit with the initial shame and guilt feelings of after it’s happened. And when you tell people and they say, well, why didn’t you talk to me? Why didn’t you call me? Why didn’t you do this? Why didn’t you do that? I’m like, you don’t get it. So I think just more understanding of that side of it, of what it feels like to be in that moment would’ve probably helped with the, it honestly would’ve helped with reaching out, I think.”

Participant 6

Young women also wanted those around them to feel empowered to reach out to support them rather than expecting the person engaging in self-harm to reach out for help. One participant noted that she would have liked her parents to facilitate her access to formal mental health support services.

“To increase awareness among parents of self-harming behaviour in young women and what it means and the importance of taking it seriously, taking the kid to a GP or a psychologist rather than misconceptions about it, because that would’ve been helpful for me.”

Participant 15

Many felt they carried the burden of educating loved ones about self-harm, which was particularly onerous due to the young women’s poor mental wellbeing at the time. Participants believed that providing loved ones with an opportunity to be educated about self-harm would reduce this burden and improve the likelihood their family and friends would respond appropriately to a disclosure of self-harm.

“I think sometimes I feel like I have to educate this person all about self-harm and why people engage in it and all that sort of thing. So just something to be like, here you go. Read this.”

Participant 3

##### Youth Specific Mental Health Education

Participants wanted more youth-specific mental health education, with the understanding that poor mental health often precipitated self-harm instances. Several participants discussed the poor state of mental health education when they were in school, and felt the lack of education increased their propensity for self-harm.

“I always think if I had had intervention earlier, I don’t believe that I would still be doing it. So I don’t know. I don’t know. I wish it was sort of more general information. I don’t think it was ever spoken about. I mean mental health education when I was growing up was awful.”

Participant 27

Young women wanted education to dispel mental health myths and normalise the experience of struggling with mental health to reduce feelings of isolation. Young women saw greater mental health education as a possible avenue to help them reduce their self-harming behaviours, or if received early enough, to eliminate the need to start self-harming in the first place. Improved access to information would also help young women perform further research to better understand their own mental health and develop their own strategies to maintain wellbeing.

“I think I would’ve just liked to see more resources about mental health while I was starting middle school, that kind of age. I think that’s when everyone sort of gets a little bit more stressed with the realities of growing up, but … none of us were really well-educated on mental health.”

Participant 9

Additionally, participants wanted information about how to access support from mental health professionals and about strategies to support themselves during challenging periods. Many wanted information about confidentiality and what information would be shared with parents, as many delayed telling their psychologists about the self-harm for fear of parents being made aware.

“I didn’t know whether they would, it’s like obviously they give the little talk at the start of if you’re at risk of harm and that doesn’t specify whether that counts and whether that means they can break confidentiality because that stopped me from telling a psychologist for a long time.”

Participant 8

##### Information About Alternative Coping Strategies

Participants reported needing more education about alternative coping strategies, noting that stopping the behaviour was only possible when effective alternative strategies had been established. One participant noted that such techniques would be more beneficial to them than trying to address the underlying triggers for the self-harm behaviour.

“More sort of active prevention tactics for in the moment from my therapist would’ve possibly been helpful rather than, of course, going into the depths of where it comes from and everything like that is incredibly important and that needed to happen as well. But combining that with active prevention tactics would’ve also probably been really helpful as well.”

Participant 6

The functions of coping strategies were extremely nuanced, and it seemed participants wanted a more bespoke approach to coping strategy suggestions. Participants sometimes spoke about alternative coping strategies they had been provided previously, which were unhelpful, leading them to feel as though their self-harm was not being taken seriously nor understood by others. Other young women found certain coping strategies helpful if the strategies were well aligned with how they were feeling (e.g., high arousal due to anxiety, or low arousal due to depression), but also provided similar physical sensations to their preferred self-harm method.

“Knowing what to do when you do want to self-harm is helpful whether you do those alternatives or not … I think it is helpful to have an understanding of, oh yeah, you get quite aroused. You can have a shower to balance that out. So those kinds of strategies are helpful I think as well.”

Participant 13

##### Would Not Want More Support

Three participants discussed not wanting support for their self-harm. Mostly, this was due to participants not perceiving that self-harm was a problem which needed to be addressed.

“…at that stage I didn’t really see it [self-harm] as a problem or anything. So, I don’t know if I would’ve wanted information about it, but maybe it would’ve been helpful. I don’t know. …”

Participant 13

One participant felt that help from others was disingenuous. She reported that she, herself, was the best person to understand her own experiences and the value that self-harm had for her. Another participant mentioned worrying that providing more information and support around self-harm may increase self-harm behaviours.

“I think in my situation there was just so much resistance and I think that a lot of information, I probably just would’ve rolled my eyes out. There was just a feeling of I know better and don’t need anything else and all that kind of thing.”

Participant 16

In summary, young women identified various types of information which would be valuable to them when they are actively engaging in self-harm, including wound management or alternative coping strategies. They also highlighted information which would have the potential to reduce their engagement in self-harm, such as improved youth mental health education. Participants also highlighted the positive impact this information could have if it were shared with those around them. However, a small number of women reported not wanting additional information or support.

#### 3.2.2. How Young Women Would Like to Receive Support for Self-Harm

##### Provided by Health Professionals

Young women spoke of wanting self-harm education and interventions to be provided by a health professional, including doctors and psychologists. Participants felt that health professionals were a trustworthy source of high-quality information or psychological support if necessary. Additionally, participants mentioned that if health professionals disseminated self-harm information or support, this would help to strengthen the perception of health professionals as people to turn to when the young women are experiencing self-harm or mental health challenges. Some participants even saw this as an approach to help mitigate some of the potential contagion risks of discussing self-harm.

“I guess it would just be that anything shared would need to be accompanied by adequate support as well. I think it’s a tough one because … it’s never going to be easy talking about all this stuff, and there’s always going to be some form of repercussion. I think it’s the support that comes alongside it, which is what would be beneficial.”

Participant 22

Participants noted that the health professional disseminating information did not need to be one they were already engaged with, with some indicating that being provided with self-harm education by any health professional would encourage them to discuss self-harm with their own doctor or psychologist.

“But it is best if you use a therapist in order to manage it [self-harm] because it is so much more controlled and you are able to trust your clinician and trust their professionalism and know that they’re delivering the information to you in a way that you can digest it and healthily be able to access the support that you need.”

Participant 17

However, participants also discussed the importance of improving the training of health professionals—particularly hospital staff and general practitioners—regarding mental health conditions and self-harm. Specifically, young women wanted health professionals to be equipped with knowledge and skills to appropriately respond to self-harm, reducing the likelihood of young women encountering invalidating interactions with health professionals. Furthermore, participants recounted having trouble accessing mental healthcare professionals when they were in distress. Increased training and support for health professionals like general practitioners would help improve access to quality support.

“I think definitely increased training on how to deal with, I think every medical specialty needs more training on how to deal with mental health. If you’re dealing with people who are sick, they’re going to be sad. You need to know how to deal with mental health. But for some reason that’s just kind of ignored. I’m studying medicine at the moment. I’m two years in; we haven’t touched on it at all.”

Participant 8

##### In the School Setting

Many young women spoke about wanting more education about self-harm as part of the school curriculum, with most suggesting it would fit into health education classes. Participants felt embedding it into the existing curriculum rather than running special seminars would increase engagement. Running special one-off seminars reinforced the notion that self-harm was unusual and increased young women’s feelings of shame and isolation in relation to their self-harm.

“I think if it was part of the wider curriculum for health and personal safety education in schools, but not done as a special topic or a seminar that you have once a year, but as just any other topic that we learn about, because I think that way people are more likely to engage with it.”

Participant 7

Participants wanted information about self-harm to help reduce feelings of embarrassment and to help them understand their experiences. Young women spoke of not knowing how to verbalise their self-harm, or not even recognising that their behaviour constituted self-harm, both of which stopped young women from reaching out for help or accessing resources. Providing such information in schools would help ensure that all young women have access to this important, validating information.

“I think that for people who are trying to hurt themselves but don’t have the words to describe it as self-harm and don’t know that it’s, it’s not a moral failing, it’s a coping method that perhaps is not the most helpful.”

Participant 7

Some participants felt that providing this information generally would be more acceptable, and that if they had been approached by someone personally, they would have felt very uncomfortable. Young women also wanted information about seeing the school counsellor and for myths about mental health and help-seeking to be dispelled.

“Making it as easy as possible and having as much information available as possible about who the psychologists are, showing that they really are nice people and that they’re not going to judge you and they’re just making it really easy to actually get in and removing as many barriers as possible.”

Participant 15

Young women did note that there were risks of exposing individuals to self-harm, but saw it as a net benefit, as peers would receive this information and be in a better position to support those who are self-harming and to feel confident to encourage them to seek help from professionals.

“I think probably a valuable way of doing it would be in school maybe and opening up conversations with peers. I think that’s where you get a lot of your support from, as a young person … most people want to talk to their friends about it rather than their family. So, I think having more conversation about it in schools and having, opening up conversation with friends.”

Participant 6

##### Physical or Digital Support

Young women spoke of wanting tangible resources that were easily accessible and discreet, such as physical written materials (e.g., posters, flyers) or online resources (e.g., website, email, mobile application). Participants described the ease of accessibility and the ability to remain anonymous as key benefits of this type of information delivery. The desire for anonymity may be due to perceived stigma about help-seeking and self-harm.

“In a way that I could access anonymously without being seen to have accessed it. So not like a poster on the wall in the locker room, but an email or a flyer, like a handout that was given to everybody or something where it doesn’t put me at risk at all to be seen to be interested.”

Participant 15

Participants also discussed the ease of sharing self-harm education or support information (particularly if available online) with friends, and noted that social media platforms such as TikTok or Instagram would facilitate the distribution of information to wider audiences. These platforms were already familiar to young women and present an existing source of self-harm information, making them an easy way of providing targeted information.

“It’s hard but effectively distributing it online on platforms where young people are going to see it, they spend most of their time online, but I know that it is hard to effectively distribute it to that kind of age bracket, cause they’re probably not looking for resources. They’re not looking for support. They’re just in the little echo chamber. But if there is a way to distribute online where young people will see it, that’s a good thing.”

Participant 20

Furthermore, young women discussed the benefit of digital platforms that could allow them to easily reach out to existing websites or support/service providers (facilitating anonymity). Young women highlighted that digital platforms had the ability to incorporate multiple modes of information delivery, such as multimedia options including video or audio alongside written information, which would facilitate information sharing.

“I’m a visual and listening learner, so having something that I can actively do and write and see and all of that sort of thing. And then listening to things as well. So, podcasts and TED Talks and music and things like that.”

Participant 6

In summary, young women discussed wanting to receive information and support from people and sources which are considered trustworthy and expert. Participants also preferred receiving information in a way that allowed them to maintain their anonymity, such as receiving information during classes at school, or via digital sources. Participants also discussed the benefits of providing generalised information and support to empower peers to support one another in relation to the self-harm.

## 4. Discussion

This qualitative study explored what support young Australian women prefer in relation to self-harm. The analysis revealed that young women want more information about self-harm, resources for the people around them, youth-specific mental health education, and information on alternative coping strategies. Young women reported wanting this information and support to come from health professionals, schools, digital sources, or physical resources.

The young women in this study outlined the importance of being provided information and education about self-harm. Access to education about mental health has been demonstrated to reduce stigma [35] and increase help-seeking behaviour [36]. A recent systematic review found that poor mental health literacy and poor knowledge of the support available for self-harm or mental health have been identified a barriers to young people help-seeking for self-harm in numerous studies [37]. Other barriers identified by the authors included negative self-perceptions, difficulty expressing distress, not recognising the seriousness of self-harm, or misguided fears about confidentiality [37]. There is potential for mental health literacy programmes to target these barriers. However, there are currently no studies evaluating the effectiveness of mental health literacy programmes for self-harm. Future research is needed to co-design and assess the efficacy of self-harm literacy programmes to ensure they adequately meet the informational needs of young people.

Participants also wanted more information about how to care for themselves after self-harming, including wound management. This is consistent with the findings of another qualitative study, which reported that young people engaging in self-harm believed that wound management education would be beneficial, especially given that many young people are unlikely to seek help or disclose their self-harm behaviour to others [38]. Such education may potentially reduce some of the risks associated with self-harm behaviour, such as infection and hospitalisation. Given that a number of young women believe self-harm behaviour is not a problem and that nothing would stop them from engaging in their self-harm behaviour, a risk minimisation approach may be advantageous. Risk minimisation approaches are effective in reducing short- and long-term implications related to substance use in young people [39,40,41], although care is needed in tailoring the approach to the type of substance and the population group [41]. A similar approach may have merit when considering different self-harm methods and injury locations. Future research should investigate the applicability of different risk minimisation strategies for self-harm.

Health professionals (including doctors and psychologists) were identified as playing important roles in delivering information about, and support for, self-harm due to their perceived trustworthiness. However, this finding contrasts with numerous studies that have found that young people prefer to access support from informal sources such as family and friends, rather than from formal sources such as health professionals [15,22,42]. This preference is attributed, in part, to concerns around confidentiality, the perception that treatment is not necessary, high costs [43], and limited access, with services often only readily available for severe risk [44]. Furthermore, in Australia, public mental health service providers are often in high demand but under-resourced, leading to long wait times and admission criteria requiring the young person to be considered at significant risk to themselves or others [44,45], and similar problems with access have been reported in other high-income countries [46,47,48]. However, the preference for professional sources of support in this study may indicate that there is reduced stigma around professional help-seeking for mental health difficulties and self-harm. It may also indicate a desire for more trustworthy and credible sources of information. This shift may be due to the plethora of problematic and misleading self-harm information available online [49]. The generation of online information on trusted websites (e.g., government websites) and digital interventions from trusted services, such as healthcare providers, might efficiently address the desire for evidence-based support while protecting young women’s desire for anonymity. Alongside this, there is a need for greater funding to improve access to mental health services for young people—indeed, there is currently a “missing middle”, a cohort of young people whose needs are too great for primary care but who are not unwell enough for state-based services [50].

The school environment was also identified as a preferred outlet to obtain information and support. Participants noted that such education would fit into the physical health curriculum, and that this could be an opportunity to remind adolescents about access to school counsellors and psychologists. McAndrew and Warne [51] reported similar findings, with young people noting that although health initiatives are offered in school, none are offered for self-harm. However, self-harm can be impacted by a contagion effect, by which individuals may be more likely to engage in self-harm behaviours after exposure to related information such as self-harm methods and imagery [52]. There is also some evidence that school-based programmes lead to increased distress in some participants [53]. As such, care should be taken to ensure that classroom-based education is free from high-risk information such as discussion of self-harm methods and that adolescents are aware of easily accessible mental health support. Furthermore, a recent review has suggested that universal school-based mental health prevention programmes are less effective than tailored approaches [54]. It may therefore be more helpful to deliver certain information (e.g., information about alternative coping strategies, information about wound management) specifically to young people identified as being at high risk of self-harm, rather than integrating this information into the general physical education curricula. Future research should focus on gathering an understanding of what specific information would be helpful to young people and what specific information poses a risk in classroom settings and should seek to evaluate the efficacy of school-based self-harm interventions. However, the implementation of school-based interventions may differ across educational and cultural contexts. For instance, private schools tend to have better financial resources compared to public schools [55] and may therefore be able to more easily implement self-harm education or support. Yet, the participants in this study indicated that health professionals were a trustworthy source of self-harm education. As such, there may be value in funding health professionals to deliver such education in schools, with the added benefit of them being an external source without a pre-existing or ongoing relationship with the students.

A small number of participants indicated that they did not want to receive support for their self-harm. Numerous studies have demonstrated that some young people do not perceive self-harm as problematic [43,56]. Self-harm has instead been reported to be an effective way of managing suicide risk [57,58] and emotional distress [59,60,61]. Young people engaging in self-harm are therefore likely to benefit from interventions addressing the source of their distress, rather than from interventions that directly aim to reduce self-harm behaviour, given that the latter may negatively impact their suicide risk.

There are some limitations to our research. First, our sample did not include any current school students, aged 16–17 years, and thus did not have input about educational activities for self-harm from young women currently attending school or from younger participants more broadly. Second, data pertaining to participant sexual orientation (e.g., LGBTIQ+ status) were not collected, and no participants identified as being Aboriginal or Torres Strait Islander. Given that these are high-risk populations for self-harm, additional investigation is required to better understand their unique support needs and preferences. Third, recruitment through social media platforms may have reduced our access to individuals who are not engaged with social media, or to the platforms we specifically utilised. Although a large proportion of Australian young people utilise social media [62], the recruitment approach utilised means that our sample was not necessarily representative of all young people engaging in self-harm. For instance, the findings may not be generalisable to young people engaging in self-harm who have presented to hospital or other health settings. Additional interviews with young women presenting to hospital or other health services for self-harm would provide a more nuanced understanding of this particular experience. We also collected limited demographic information to maintain participant anonymity; however, we acknowledge that this also limits the generalisability of our findings. Our recruitment approach may have also influenced young women’s interest in digital resources due to existing familiarity and comfort in utilising such resources. Our study also had a number of strengths. First, the robust sample size of 27, which is comparatively larger than other qualitative self-harm studies, and the systematic approach to data analysis both allowed for detailed analysis and a greater confidence in our resulting themes. Second, the co-design of the interview guide with individuals with a lived experience of self-harm and individuals working in research and clinical practice ensured that the interview questions were appropriate and meaningful. Third, the online recruitment and data collection methods enabled the participation of young women from across Australia, providing a broader perspective on self-harm support preferences.

## 5. Conclusions

The current study provides insight into the type and format of support that is preferred by young women engaging in self-harm. We found that young women want information about self-harm and mental health, coping strategies, and resources for those around them, and that they prefer to receive this information from health professionals, schools, online sources, or written resources. The findings highlight the need to improve access to formal mental health support among young people, the importance of young people being able to access support confidentially, and the need for those around young people to be educated about self-harm. Additionally, the findings support a role for schools in providing information about mental health and available support, although further research is needed to evaluate the efficacy of such school-based interventions.

## Figures and Tables

**Table 1:** Participant demographic information.

Demographic		N (%)
Employment status	Employed part-time	21 (77.8%)
	Unemployed	6 (22.2%)
Student status	Student full-time	10 (37.0%)
	Student part-time	8 (29.6%)
	Not studying	9 (33.3%)
State or territory of residence	NSW	9 (33.3%)
	QLD	7 (25.9%)
	VIC	5 (18.5%)
	SA	2 (7.4%)
	TAS	2 (7.4%)
	Other *	2 (7.4%)
Mental health diagnosis **	Depressive disorder	21 (77.8%)
	Anxiety disorder	17 (63.0%)
	Feeding and eating disorder	7 (25.9%)
	Neurodevelopmental disorder	7 (25.9%)
	Obsessive–compulsive disorder	4 (14.8%)
	Trauma-related disorder	4 (14.8%)
	Other disorder	6 (22.2%)

Note: * other was used where state or territory of residence *n* = 1, ** many participants reported multiple mental health diagnoses.

**Table 2:** Theme structure.

Theme	Code
3.2.1. What support young women want around self-harm	Information about self-harm
	Information about injury management
	Information for the people around them
	Youth-specific mental health education
	Information about alternative coping strategies
	Would not want more support
3.2.2. How young women would like to receive support for self-harm	Provided by health professionals
	In the school setting
	Physical or digital support

## Data Availability

The data presented in this study are available on request from the corresponding author due to ethical considerations.

## Supplementary Materials

The following supporting information can be downloaded at: https://www.mdpi.com/article/10.3390/ijerph22040587/s1, Supplementary S1: Interview Guide.
